# Rheological Property Changes in Polyacrylamide Aqueous Solution Flowed Through Microchannel Under Low Reynolds Number and High Shear Rate Conditions

**DOI:** 10.3390/mi16050545

**Published:** 2025-04-30

**Authors:** Yishuai Li, Yukihiro Yonemoto, Yuki Yamahata, Akimaro Kawahara

**Affiliations:** Faculty of Advanced Science and Technology, Kumamoto University, Kumamoto 860-8555, Japan; 234d9286@st.kumamoto-u.ac.jp (Y.L.); 244d9282@st.kumamoto-u.ac.jp (Y.Y.); akimaro@mech.kumamoto-u.ac.jp (A.K.)

**Keywords:** microchannel, microfluidics, rheological properties, polymer solution, viscosity, relaxation time, polyacrylamide

## Abstract

As an important structure of microfluidic devices, microchannels have the advantages of precise flow control and high reaction efficiency. This study investigates experimentally changing the rheological properties of a polyacrylamide (PAM) aqueous solution after flowing through a square microchannel with a hydraulic diameter of 0.5 mm under low Reynolds number and high shear rate conditions. To know the effect of the channel length on the change in viscosity and relaxation time, the length is changed to 100 mm and 200 mm. From the experiment, it is found that both the viscosity and relaxation time of the solution decrease with increasing the shear rate and the microchannel length. Based on the present experimental data, an empirical model is proposed to predict the change ratio of the relaxation time before and after passing through the microchannel, and the calculation with the model has an agreement with the experiment with root-mean-square absolute error of 0.007.

## 1. Introduction

Flow characteristics in a microchannel with a hydraulic diameter of less than 1 mm have been receiving significant attention from many researchers over the past decades [1,2,3,4,5,6]. As the channel dimension decreases, the influence of inertial force on the fluid flow continues to decrease, which makes fluid regulation possible by controlling the surface and the flow rate of the fluid. By precisely controlling the flow rate of fluids in microchannels, cell separation [7] and dynamic control of the cell microenvironments [8] can be achieved.

Polyacrylamide (PAM), as a high molecular polymer commonly used in biochemistry, is widely used to make solutions or hydrosols for transporting drugs or fixing proteins, etc. [9,10,11]. PAM aqueous solutions have a long chain structure of molecules, and their rheological properties, such as viscosity and relaxation time, will change during flow in a channel. This change is called mechanical deterioration since the viscosity and the relaxation time usually tend to decrease. Such degradation is due to the stretching of the polymer molecular chains under the action of stress, which leads to fracture of the chain structure.

At present, the research on the degradation of polymer solutions focuses on two aspects. One is the degradation at the flow contraction under a low Reynolds number (a laminar flow) condition, and the other is the turbulence under a high Reynolds number (a turbulent flow) condition. In a laminar flow, due to the predominance of viscous force, the streamlines are evenly distributed, and the polymer molecules are arranged along the direction of shear force. At the sudden contraction of the cross-section in the flow channel, the polymer molecules begin to be fractured under the action of the normal force [12,13,14,15,16,17,18,19]. The main points of view on the causes of turbulence degradation are divided into two categories. The first one is that polymer molecules are stretched by the turbulent structures [20,21,22]. The second one is that under specific flow conditions, the turbulent kinetic energy is transformed by a counter-torque-like mechanism into elastic energy, some vortices are destroyed, and the elastic energy is released to the mean flow [23,24,25,26,27].

Typically, the flow in a microchannel is a laminar flow with a smaller Reynolds number and a higher shear rate. Recent reports on the elastic instabilities of polymer solution in microchannels offer a possible mechanism for polymer molecular degradation at low Reynolds numbers [28,29,30]. Even under low Reynolds number conditions, small disturbances in the polymer solution can amplify this nonlinear instability due to elastic stresses, resulting in turbulence similar to that observed under high Reynolds number conditions. Such elastic turbulence depends only on the magnitude and duration of elastic stress. Usually, elastic turbulence can be generated when the Weissenberg number (Wi) is greater than 5.1 [31]. Although there is much debate about the mechanism of degradation caused by elastic turbulence, as more and more polymer solutions are applied in biology and chemistry, the estimation of the changes in the rheological properties of polymer solutions have become increasingly important. For example, in drug delivery systems using polymer solutions [9,10,11] the drug release rate can be adjusted by controlling the viscosity and relaxation time.

There are models [32] that can evaluate the rheological properties of a polymer solution before use, i.e., before it is passed through the flow channel, based on its molecular weight. On the other hand, it is difficult to predict the change in the rheological properties after passing through a flow channel because it is hard to quantify each factor directly that affects the property changes. One of the design guidelines for a microreactor is the length of the flow channel because the length is related to the residence time of a solution in the reactor, which affects the reaction rate and/or efficiency [33,34]. Therefore, the size of the flow channel could become an important measure for the evaluation of the change in the rheological properties.

In this connection, the purpose of this study is to develop an empirical model for indirectly and simply evaluating the change in the rheological property of polymer solution flowed through a microchannel. In this paper, we will investigate experimentally the change in rheological properties of a PAM solution after flowing in a microchannel with different lengths of flow channel under a low Reynolds number and high shear rate conditions. By letting the solution flow through square microchannel with sides of 0.5 mm, the viscosity and relaxation time of the solution flowed in the channel are measured. By comparing the viscosity and relaxation time before and after flowing through the microchannel, the effects of shear rate and channel length on their rheological properties are considered. And then, an empirical model is proposed to predict the relaxation time from the flow rate of the solution and the microchannel length.

## 2. Experiment

### 2.1. Test Fluids

Flexible polymers are used in the present experiment, i.e., polyacrylamide (PAM) (Polysciences, Inc., Warrington, PA, USA, molecular weight, $M_{w}$ = 6,000,000 g/mol). The critical overlap concentration $c^{*}$ is defined as the concentration at which the polymer coils start to overlap with each other, and $c^{*}=1/\left[\eta \right]$ [35], [η] [-] is the intrinsic viscosity of the solution, and it is estimated by using the Mark–Houwink–Sakurada equation [36]:

(1) $$\left[\eta \right]=0.00742M_{w}^{0.775}$$

In this study, the maximum value for $c^{*}$ of PAM is 753 ppm. Maerker et al. [17] found that a mechanical degradation appears to be very nearly independent of the PAM concentration between 300 and 600 ppm in a salt solution of 3.0% and 3.3% total dissolved. Thus, to better demonstrate the mechanical degradation phenomenon, two semi-dilute solutions of PAM are prepared, with its concentration of 0.1 wt% (PAM 0.1) and 0.3 wt% (PAM 0.3) in pure water. Since the concentration of the prepared solution *c* is larger than $c^{*}$, the polymer chains in the solution are entangled and there is interaction and friction between them. Considering that stirring to dissolve the polymer in pure water may affect the mechanical properties of the solution, three kinds of test liquids dissolve naturally at rest under a room temperature ($24^{\circ }$) to avoid a degradation during the preparation process.

### 2.2. Test Apparatus and Flow Conditions

**Figure 1:**
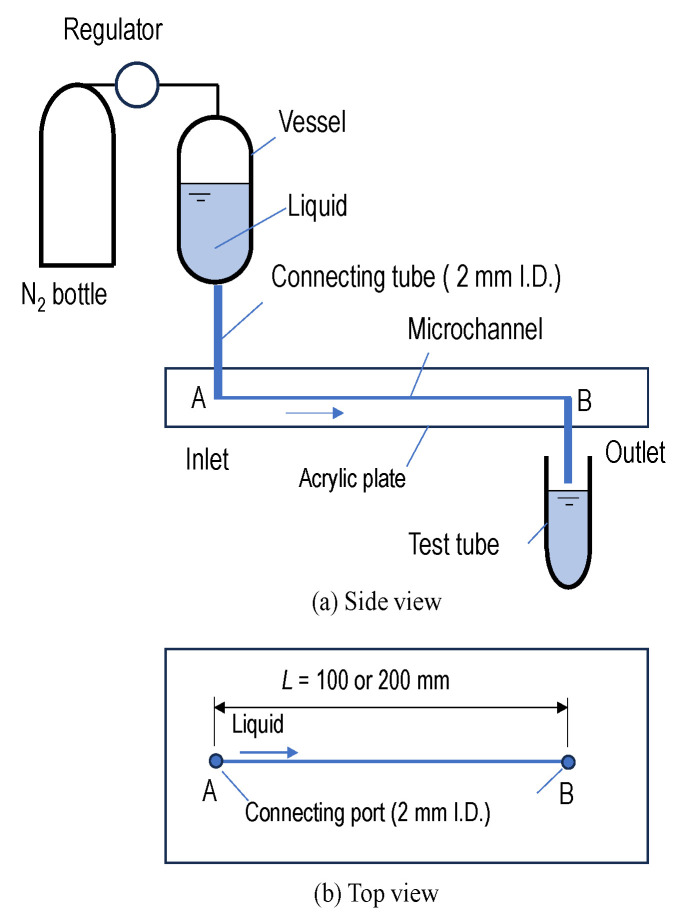
Schematic of experimental apparatus.

Figure 1 shows a schematic diagram of the test apparatus. The test microchannel is fabricated by two acrylic plates. The flow channel is created by overlapping a grooved plate with another flat one. The cross-sectional geometry of the channel is a square with one side of 0.5 mm. The test liquid is stored in a vessel. The liquid is supplied from port A and flowed through the microchannel. The volume flow rate of the liquid supplied is adjusted by regulating the pressure of nitrogen in the vessel as shown in Figure 1a. The liquids are discharged into the atmosphere from port B and are collected into a test tube. The rheological properties of the collected liquids are measured with the method described in Section 2.3. In order to avoid unnecessary mechanical degradation of the liquids before entering the microchannels as much as possible, the pipes before entering the microchannels are made of 2.0 mm (inner diameter) plastic tubing with a length of 100 mm.

As shown in Figure 1b, the length of the microchannel, *L*, is 100 mm and 200 mm. At the same flow rate, the liquids flow residence time in the 200 mm long microchannel will be twice as long as in the 100 mm one. This is a critical experimental design because the experimental results will reflect the role of elastic turbulence in the mechanical degradation process. If the mechanical degradation depends only on extensional flow through contraction (such as flowing through the inlet of the microchannel), the experimental results will be independent of the flow time in the microchannel.

The shear rate is used here as the velocity gradient in the microchannel. This consideration is based on two main points. Firstly, in the engineering point of view, one often needs to consider the Reynolds number and Weissenberg number to evaluate the flow condition of the fluid. For viscoelastic fluids such as polymer solutions, the apparent viscosity is a function of the shear rate. The use of the shear rate allows for a better assessment of the degree of degradation induced in the present microchannel. Secondly, for flow paths such as channels and tubes, the rate of extension in the face of contraction or expansion is a function of the shear rate [37]. In present study, the shear rate in microchannel is defined as follows:

(2) $${\dot{\gamma }}_{mc}=8u/D_{H}$$

Here, *u* [m/s] is the average velocity of liquid in the microchannel, and $D_{H}$ [m] is the hydraulic diameter of the microchannel. The average velocity is determined by dividing the volume flow rate by the cross-sectional area of the channel.

The experimental liquids flow through the microchannel under shear rate conditions: ${\dot{\gamma }}_{mc}=$ 6000, 10,000, 20,000, 40,000, 60,000, 80,000 and 100,000 1/s. For all shear rate conditions, the Reynolds number range of the experimental environment is 100 to 1800, and the Reynolds number Re is defined as

(3) $$Re=\frac{\rho uD_{H}}{\eta }$$

where ρ [${\mathrm{kg/m}}^{3}$] is the density, and η [Pa· s] is the viscosity. In order to ensure that the degradation occurs inside the microchannel, the corresponding conditions are estimated using the Weissenberg number Wi, defined as

(4) $$Wi={\dot{\gamma }}_{mc}\lambda$$

where λ [s] is the relaxation time. In this study, the minimum value of Wi in the microchannel is 44.3. According to the research of Pan et al. [31], the subcritical nature of the transition has been reached, and turbulence caused by elastic stress exists.

### 2.3. Measurement Methods for Rheological Characteristics

The shear rheological measurements in this study are performed using TA instruments Discovery Hybrid Rheometer-2 with a cone and plate geometry at a fixed temperature 20 °C. The uncertainty in the viscosity data is estimated to be ±2% [38,39].

**Figure 2:**
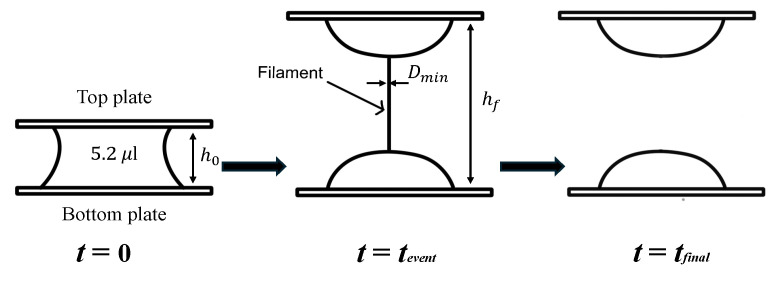
Sketch of extensional elasto-capillary thinning measurement. When the liquid evolves into a filament, the minimum diameter $D_{min}$ is recorded.

An extensional elasto-capillary thinning measurement method is used to measure relaxation time for evaluating the extensional rheological properties [40,41]. As shown in Figure 2, a small quantity of the test liquid sample (5.2 μL) is placed between two plastic plates made of polycarbonate. The initial separation height which is between two plates, $h_{0}$, is set to be 1.22 mm (*t* = 0). The liquid confined between the two plates is stretched as the top plate is moved linearly to a final height $h_{f}$ = 2.44 mm. As shown in Figure 2, a cylindrical liquid filament is formed between two plates, and the diameter of the filament, $D_{min}$ [m], becomes thinned exponentially in time, and the filament eventually breaks (*t* = $t_{final}$). During the thinning process of the filament, the minimum diameter of the filament is recorded by a high-speed video camera (MEMRECAM HX-3, Nac Image Technology Inc., Tokyo, Japan) under 2000 FPS.

**Figure 3:**
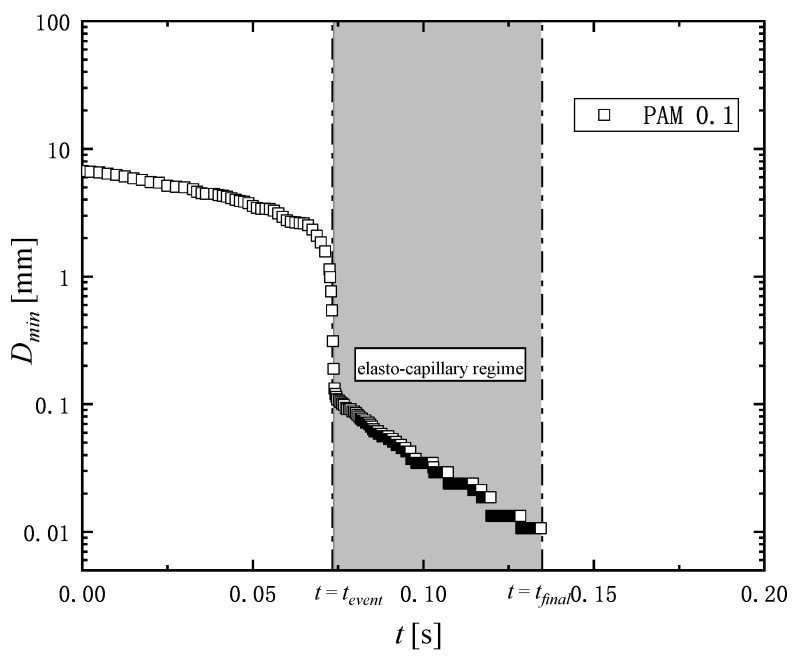
Typical example of time variations in liquid filament diameter of PAM 0.1 solution in capillary thinning measurement. The $t_{event}$ is the start time of the elasto-capillary thinning regime, and $t_{final}$ indicates the end time at which the filament breaks, and the relaxation time is captured in the elasto-capillary regime.

Figure 3 shows an example of the measurement of the change in diameter of a liquid filament over time. The thinning dynamics of the filament is driven by capillary forces. The capillary thinning is resisted by a combination of the viscosity, inertia, and elasticity of liquids, which depends on the liquid’s physical properties. The competition among the inertia, viscosity, and elasticity during the filament thinning process gives rise to three distinct filament breakup regimes known as the inertia-capillary, the visco-capillary, and the elasto-capillary regime [42,43]. In the elasto-capillary regime over $t=t_{event}$ to $t=t_{final}$ as shown in Figure 3, the filament is in an elasto-capillary equilibrium and continues to thin due to capillary forces until the filament breaks at $t=t_{final}$.

In the extensional elasto-capillary thinning measurement, the relationship between the minimum diameter $D_{min}$ of the filament and the event time during this process can be described by the exponential decay function [44,45]

(5) $$D_{min}=Cexp^{-\frac{t_{event}}{3\lambda }}$$

where *C* [m] is the fluid-dependent constant, $t_{event}$ [s] is the time of the elasto-capillary thinning regime, and λ [s] is the relaxation time. The relaxation time can be determined from the diameter of the filament in the capillary thinning process by fitting Equation (5) to the experimental data as shown in Figure 3. The relaxation time is used to evaluate influence of degradation in the extensional rheology.

## 3. Result and Discussion

### 3.1. Shear Rheology

**Figure 4:**
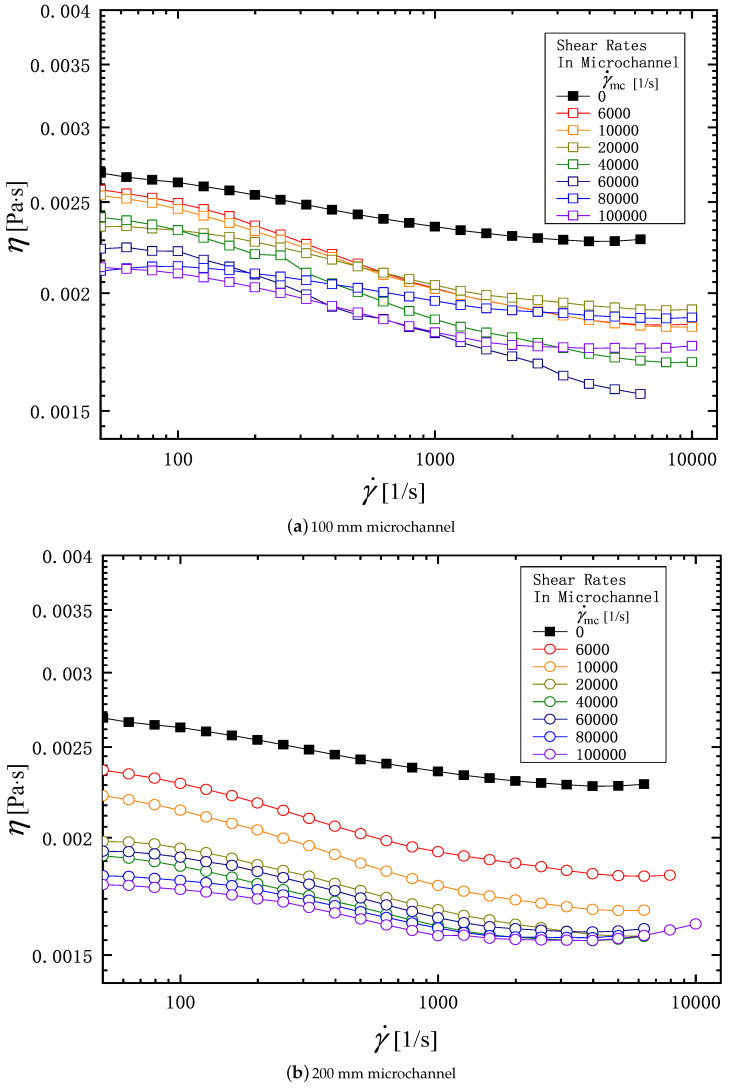
Effects of the shear rate in microchannel ${\dot{\gamma }}_{mc}$, and the channel length on viscosity profile of the PAM 0.1 solution. (**a**) 100 mm microchannel; (**b**) 200 mm microchannel.

**Figure 5:**
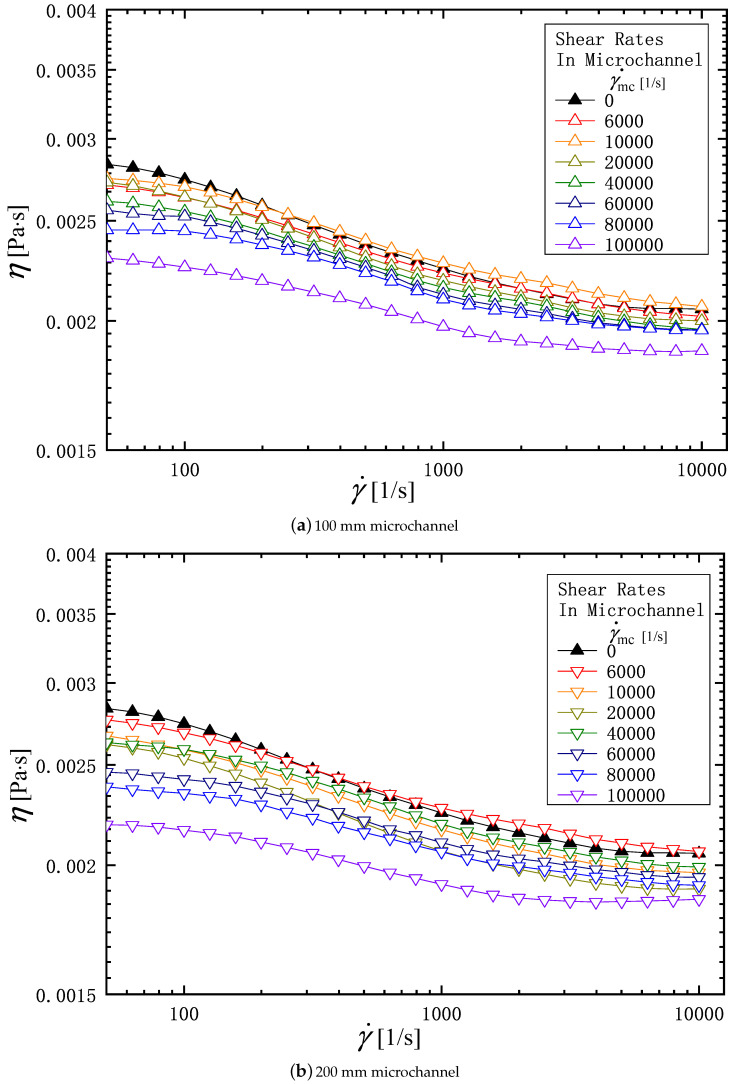
Effects of the shear rate in microchannel ${\dot{\gamma }}_{mc}$, and the channel length on the viscosity profile of the PAM 0.3 solution: (**a**) 100 mm microchannel; (**b**) 200 mm microchannel.

Figure 4 and Figure 5 show, respectively, the viscosity of the PAM 0.1 wt% and 0.3 wt% aqueous solution measured with a rheometer before and after it is passed through the microchannel. In each figure, subfigures (a) and (b) show the viscosity through 100 mm and 200 mm long microchannels, respectively. The ordinate is the viscosity, and the abscissa is the shear rate in rheometer, $\dot{\gamma }$ [1/s]. The symbols in each figure are distinguished by the shear rate, ${\dot{\gamma }}_{mc}$, when the solution is flowed through the microchannel. Therefore, the solid symbol (${\dot{\gamma }}_{mc}=0$) indicates the value before the solution is flowed through the microchannel. In the viscosity measurement, each sample is measured three times, and the average value is taken to draw the curve in these figures. The deviation of each measurement from the average value is within 5 %. Regardless of the solution concentration (0.1 w% or 0.3 wt%) and the microchannel length (*L* = 100 mm or 200 mm), all the liquids tested exhibit pseudoplastic (or shear thinning) behavior, in which the viscosity decreases with an increasing shear rate in rheometer, $\dot{\gamma }$. For PAM 0.1 wt% solution, focusing on the relationship between the viscosity of a solution and the shear rate in a microchannel ${\dot{\gamma }}_{mc}$, we can see that the viscosity decreases with the increasing of ${\dot{\gamma }}_{mc}$ at the fixed rheometer shear rate, $\dot{\gamma }$. In addition, comparing subfigures (a) and (b), we can see that even at the same ${\dot{\gamma }}_{mc}$, the viscosity decreases as the length of the channel increases. A longer channel means that the polymer has a longer residence time within the channel and is subjected to longer shear forces. The results for the PAM0.3 solution shown in Figure 5 show the same trend as that for the PAM0.1 solution.

Here, the power-law model, which expresses viscosity of non-Newtonian fluid, is defined as

(6) $$\eta =K{\dot{\gamma }}^{n-1}$$

**Figure 6:**
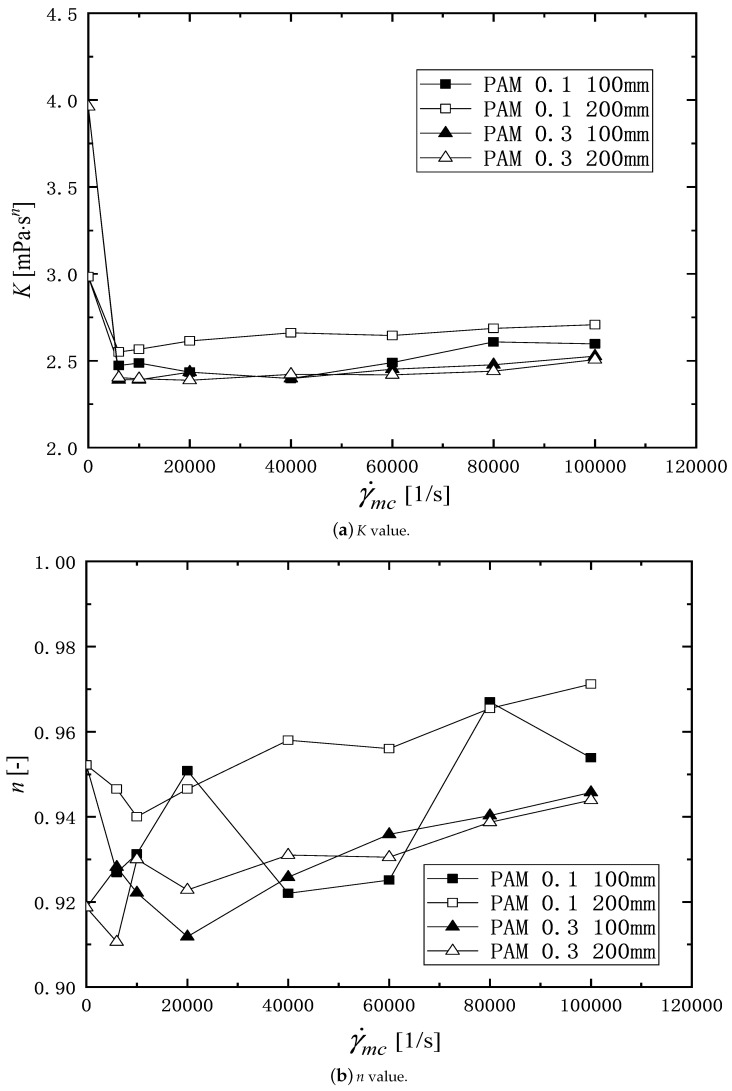
Effects of the shear rate in microchannel ${\dot{\gamma }}_{mc}$, and the channel length on the value of *K* and *n* in the power-law model of non-Newtonian viscosity. (**a**) Pseudo-plastic viscosity, *K*; (**b**) flow behavior index, *n*.

In Equation (6), *K* [Pa· $s^{n}$] is the pseudo-plastic viscosity, *n* [-] is the flow behavior index, and $\dot{\gamma }$ [1/s] is the shear rate in rheometer. Table 1 shows the values of *K* and *n* obtained from the viscosity profile data in Figure 4 and Figure 5. Figure 6a,b show the *K* and *n* values plotted against the shear rate, ${\dot{\gamma }}_{mc}$. The *K* values of the PAM solution after passing through the channel are smaller than the value at ${\dot{\gamma }}_{mc}=0$ and are almost constant against the shear rate, ${\dot{\gamma }}_{mc}$. Although there is some scatter, the value of *n* tends to increase with the increasing of ${\dot{\gamma }}_{mc}$, indicating that the pseudo-plasticity decreases.

The degradation of viscosity is reflected in the reduction in the viscosity. In order to visualize the degree of the degradation at each shear rate in the microchannel ${\dot{\gamma }}_{mc}$, viscosity degradation ratio $K_{\eta }$ [-] is defined as

(7) $$K_{\eta }=1-\frac{\eta_{D}}{\eta_{o}}$$

Here, $\eta_{D}$ [Pa· s] is the viscosity of the solutions after passing through the microchannel, and $\eta_{o}$ [Pa· s] is the original viscosity of solutions, i.e, the viscosity before the flowing microchannel, i.e, ${\dot{\gamma }}_{mc}$ = 0 (plotted by solid symbols in Figure 4 and Figure 5).

**Figure 7:**
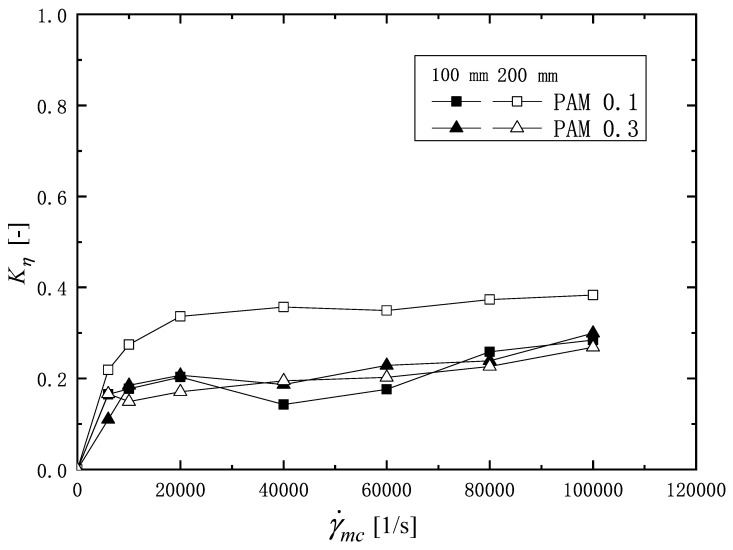
The relationship of viscosity degradation factors $K_{\eta }$ with shear rate in microchannels ${\dot{\gamma }}_{mc}$, open symbol for 200 mm microchannel, and solid one for 100 mm microchannel.

Figure 7 shows the viscosity degradation ratio $K_{\eta }$, plotted by the shear rate in the microchannel ${\dot{\gamma }}_{mc}$. The square and triangular symbols indicate the PAM 0.1 wt% and 0.3 wt% solutions, respectively. The solid and open symbols show $K_{\eta }$ values, respectively, for the microchannel length of 100 mm and 200 mm. From Figure 7, it is found that there is a region ($0<{\dot{\gamma }}_{mc}<10,000$ 1/s) where the value of $K_{\eta }$ increases rapidly with the increasing shear rate, regardless of the PAM concentration and length of the channel. Beyond this region, the value of $K_{\eta }$ shows a tendency to increase slightly. PAM 0.1 wt% solution is subjected to stronger degradation when flowing through the 200 mm microchannel than the 100 mm microchannel. The reason that more severe degradation occurs in a longer microchannel may be the asymmetric velocity distribution in the microchannel [46], which causes excessive local stress, inducing fracture of the molecular chain. As the chains are fractured, the average molecular weight of the solution decreases [14], which in turn shows a reduction in the viscosity. As for the PAM 0.3 wt% solution, the value of $K_{\eta }$ for the 100 mm long microchannel is approximately equal to that for the 200 mm long microchannel. Therefore, for the PAM solution, increasing the concentration of the polymer in the solution helps to reduce the effect of the flow residence time on the change in viscosity and improves the ability to resist the viscosity degradation.

### 3.2. Extensional Rheology

According to kinetic theory [47], the relaxation time λ of a solution is controlled by its solvent viscosity because the viscosity of the solvent affects the elongate rate of polymer molecular chains in an extensional experiment [47]. Low solvent viscosity will affect the measurable relaxation time of liquids, and increasing the solvent viscosity will result in a higher relaxation time for the entire solution, which is consistent with the experimental results of Zell et al. [48]. Considering that the high viscosity of solution will affect its fluidity in the microchannel, only tap water is used as the solvent in this study.

**Figure 8:**
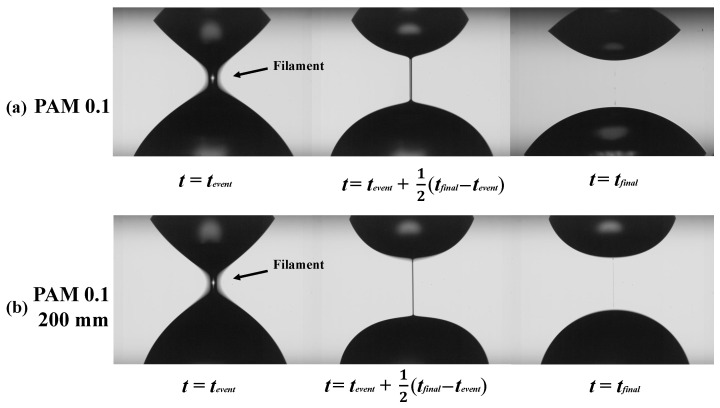
Typical example of liquid filament thinning behavior of PAM 0.1 solution in elasto-capillary regime observed by high-speed camera in extensional measurement; (**a**) before flowing through the channel, ${\dot{\gamma }}_{mc}$ = 0; (**b**) after flowed through 200 mm long microchannel with ${\dot{\gamma }}_{mc}$ = 100,000 1/s.

Figure 8a,b show the typical example of the filament thinning behavior of PAM 0.1 wt% solution in the extensional measurement described in Section 2.3. Figure 8a shows the behavior of the solution before passing through the microchannel, and Figure 8b shows the behavior of the solution passed through the microchannel with the length of 200 mm. As shown in Figure 3, $t=t_{event}$ means the start time of the elasto-capillary process, and $t=t_{final}$ the time at which the filament breaks. The duration of the elasto-capillary process, i.e., $t_{final}-t_{event}$, for ${\dot{\gamma }}_{mc}=0$ and ${\dot{\gamma }}_{mc}=100,000$ 1/s are 64 ms and 19 ms respectively. Thus, the elasto-capillary process time becomes shorter after passing through the microchannel.

**Figure 9:**
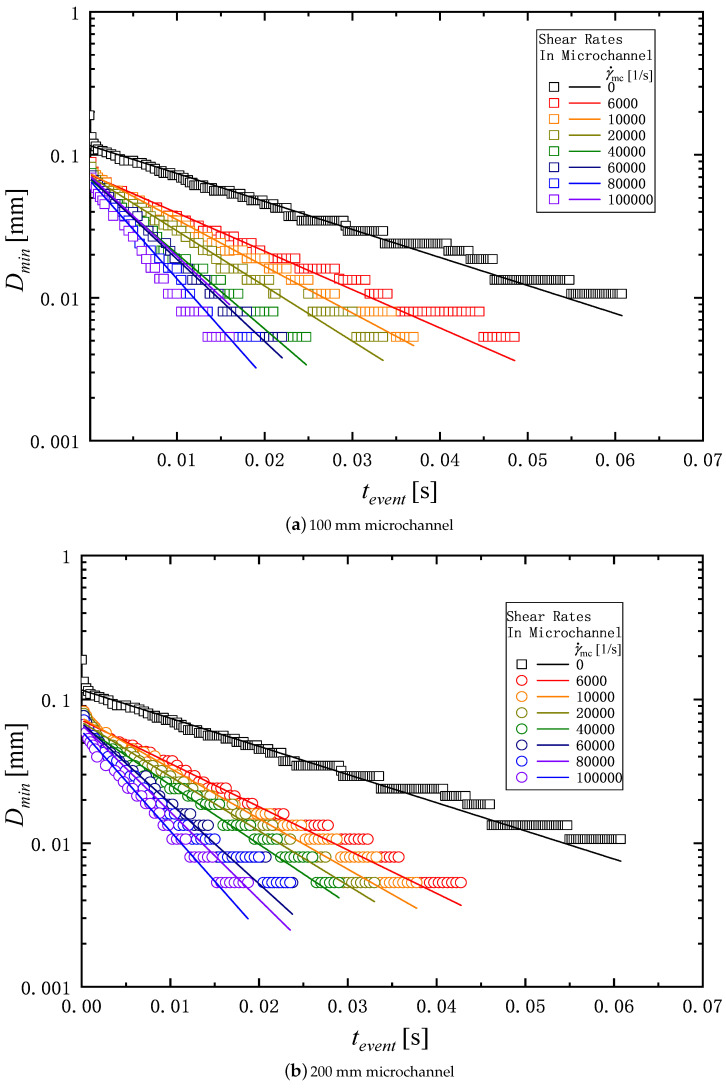
Time variations in liquid filament diameter of PAM 0.1 over the elasto-capillary regime in extensional experiment: (**a**) 100 mm microchannel; (**b**) 200 mm microchannel.

**Figure 10:**
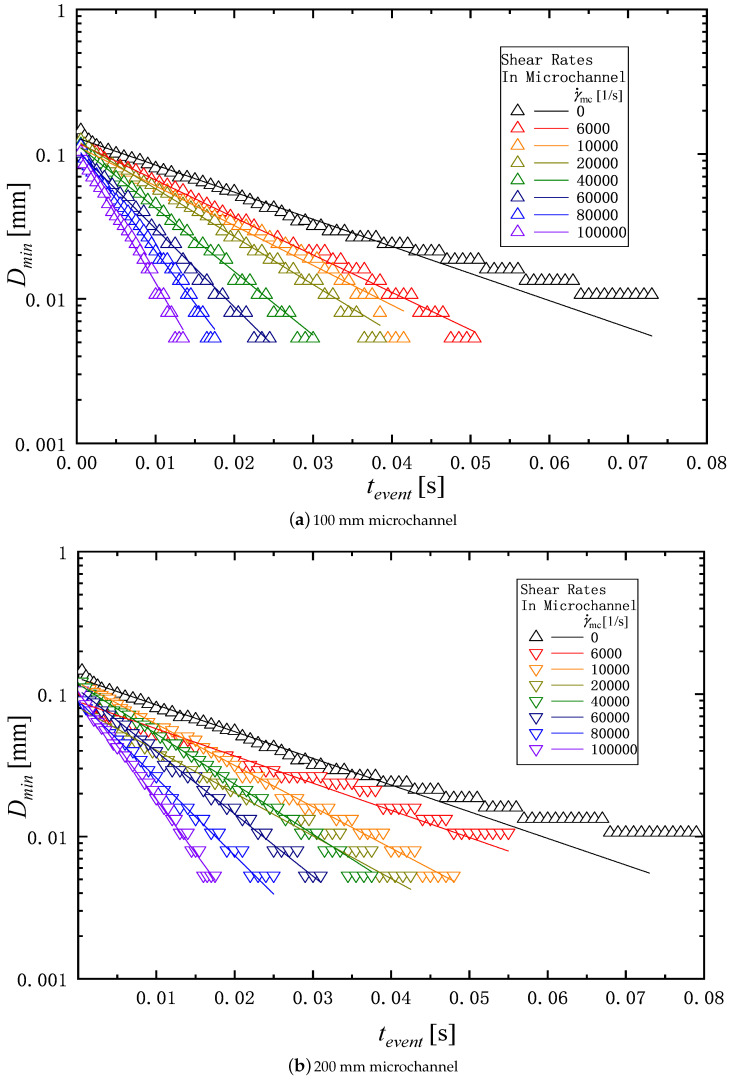
Time variations in liquid filament diameter of PAM 0.3 over the elasto-capillary regime in extensional experiment: (**a**) 100 mm microchannel; (**b**) 200 mm microchannel.

Figure 9 and Figure 10 show the filament diameter, $D_{min}$ for PAM 0.1 and PAM 0.3 as an exponential function of the time change in the elasto-capillary thinning process. The total time of the filament thinning process is decreased as the shear rate in the microchannel ${\dot{\gamma }}_{mc}$ is increased. The relaxation time is determined by fitting the data of $D_{min}$ in Figure 9 and Figure 10 to Equation (5). Table 2 shows the relaxation time determined for the PAM solutions corresponding to shear rate ${\dot{\gamma }}_{mc}$ and the length of the microchannel *L*.

**Figure 11:**
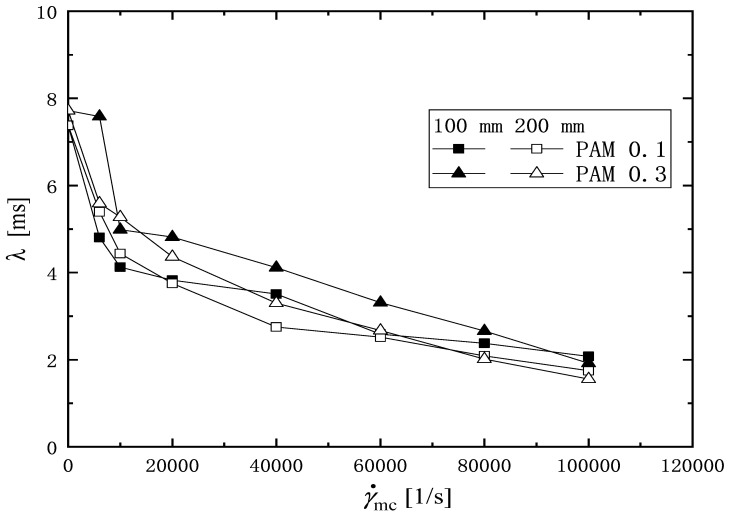
Relaxation time of PAM solution obtained under shear rate in the microchannel ${\dot{\gamma }}_{mc}$.

Figure 11 shows the relaxation times of the PAM solution against the shear rate ${\dot{\gamma }}_{mc}$ in the microchannel with different channel length *L*. Each sample is repeated twice in the extensional measurement, and the average of the relaxation times is plotted in this figure. For all data, the deviation of the maximum and minimum values from the average value is within 9%. It can be seen that the relaxation time decreases with increasing the shear rate ${\dot{\gamma }}_{mc}$, regardless of the PAM concentration and the length of the channel. The relaxation time reduces sharply over the shear rate range of $0<{\dot{\gamma }}_{mc}<10,000$ 1/s. A similar rapid reduction tendency of the average molecular weight was also observed by Vanapalli [49].

To quantitatively evaluate the change in relaxation time of the PAM solution after flowing through the microchannel, the following elastical degradation ratio $K_{\lambda }$ [-] is defined as

(8) $$K_{\lambda }=\frac{\lambda_{o}-\lambda_{D}}{\lambda_{o}}$$

**Figure 12:**
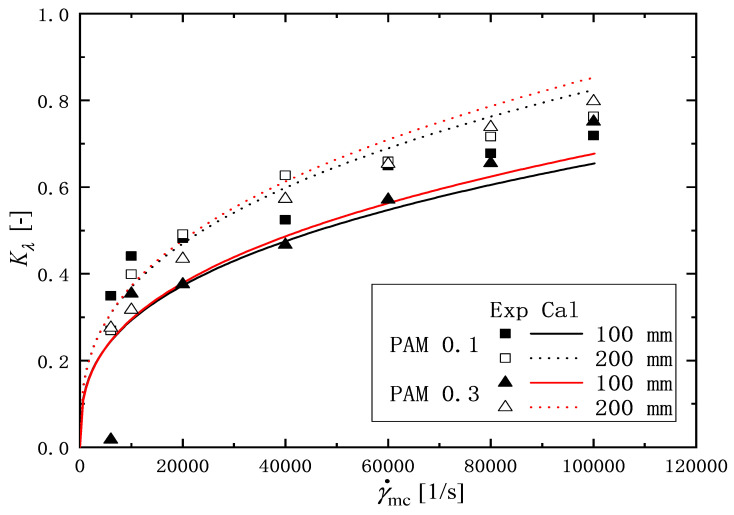
Elastical degradation ratio $K_{\lambda }$, against shear rate in microchannel ${\dot{\gamma }}_{mc}$. Two symbols and two curves show, respectively, the experimental values and the calculation one by Equation (11).

Here, $\lambda_{D}$ [s] is the relaxation time of the solutions after degradation with various shear rates in microchannel ${\dot{\gamma }}_{mc}=6000,10,000,20,000,40,000,60,000,100,000$ 1/s, $and\;\lambda_{o}$ [s] is the relaxation time of the original ones without flow in the channel ${\dot{\gamma }}_{mc}=0$. Figure 12 shows the variation of the elastical degradation ratio $K_{\lambda }$, with the shear rate in the microchannel ${\dot{\gamma }}_{mc}$. Two symbols represent the experimental values of $K_{\lambda }$, and two curves indicate the calculation mentioned in Section 3.3. For the solutions of PAM, the value of $K_{\lambda }$ increases with increasing the shear rate. In the shear rate range ($0<{\dot{\gamma }}_{mc}<10,000$ 1/s), the value of $K_{\lambda }$ increases rapidly with the shear rate. Beyond the shear rate range, the value of the ratio increases slowly with an increasing shear rate. Focusing on the effect of channel length on the relaxation ratio, it can be seen that the value of $K_{\lambda }$ for 200 mm long is greater than that of the 100 mm one. As mentioned in the introduction, factors that affect the change in the rheological properties of a solution before and after it is passed through a microchannel include the shape of the inlet and outlet of the channel (contraction and/or expansion), the length of the channel, etc. Meanwhile, in this experiment, the length of the channel is changed, but the shape of the inlet and outlet is the same, so it is assumed that the difference in the observed relaxation between 100 mm and 200 mm long channel under the same shear rate is due to the difference in the length of the channel.

### 3.3. Prediction of Relaxation Time

In this section, we discuss a simple method to evaluate the relaxation time change based on the experimental results. According to the results in Figure 12, it is found that the relaxation time change depends on the shear rate ${\dot{\gamma }}_{mc}$ and the flow channel length *L* in the microchannel. The shear rate depends on the volume flow rate *Q* [$m^{3}$/s], which is expressed as the cross-sectional area of the flow channel *A* [$m^{2}$] multiplied by the average velocity *u* [m/s]. Therefore, the flow rate, *Q*, and the size of the microchannel would be important factors for the change in the relaxation time. Thus, we assume that the characteristic lengths for the effects of the flow rate and the size of microchannel are defined by $l_{flow}={\left(Qt_{c}\right)}^{1/3}$ and $l_{sys}={\left(AL\right)}^{1/3}$, respectively. Here, $t_{c}$ [s] is the characteristic time defined as $\rho L^{2}/\eta_{o}$. From the experimental observation, it is assumed that the ratio of the change in relaxation time $K_{\lambda }$ is proportional to the ratio of the characteristic size of the $l_{sys}$ and $l_{flow}$ as follows:

(9) $$\frac{l_{flow}}{l_{sys}}\approx B\frac{\lambda_{o}-\lambda_{D}}{\lambda_{o}}=BK_{\lambda }$$

Here, $\lambda_{o}$ [s] is the relaxation time of the original solutions. *B* [-] is an arbitrary constant. For the liquid viscosity, the power-law model is used as follows:

(10) $$\eta_{o}=K_{o}{{\dot{\gamma }}_{mc}}^{n_{o}-1}$$

where $K_{o}$ [Pa· $s^{n_{o}}$] and $n_{o}$ [-] are the pseudo-plastic viscosity and flow behavior index in Equation (6) from the original solutions ${\dot{\gamma }}_{mc}=0$, listed in Table 1. By the simple transformation of Equation (9) with Equation (10), it leads to the following relation:

(11) $$K_{\lambda }=\frac{1}{B}{\left(\frac{\rho D_{H}L}{8K_{o}}\right)}^{\frac{1}{3}}{{\dot{\gamma }}_{mc}}^{\frac{2-n_{o}}{3}}$$

Here, ρ [${\mathrm{kg/m}}^{3}$] is the density, $D_{H}$ [m] is the hydraulic diameter of the microchannel, and *L* [m] is the length of the microchannel.

Equation (11) is fitted to the experimental values for PAM aqueous solutions in Table 2 and the value of *B* is determined as 109. Figure 12 shows the comparison of the experimental value for the relaxation time $\lambda_{D}$ with the value calculated by Equation (11). The calculated values have an agreement with the experimental values with the mean absolute error $\varepsilon_{ABS,M}=0.005$, and the RMS absolute error $\varepsilon_{ABS,RMS}=0.007$, whose errors are defined as follows:

(12) $$\varepsilon_{ABS,M}=\frac{1}{N}\sum_{i=1}^{N}\left(K_{\lambda_{cal}}-K_{\lambda_{exp}}\right)$$

(13) $$\varepsilon_{ABS,RMS}=\sqrt{\frac{1}{N-1}\sum_{i=1}^{N}{\left(K_{\lambda_{cal}}-K_{\lambda_{exp}}\right)}^{2}}$$

where *N* is the number of data points.

**Figure 13:**
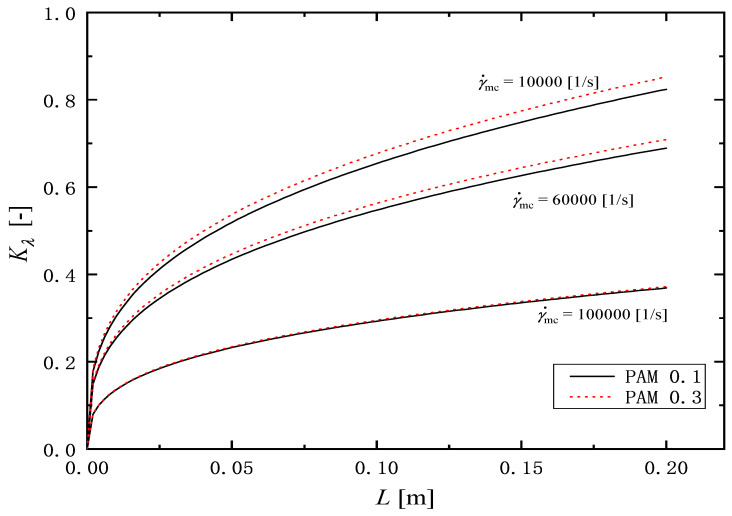
Example for estimating elastical degradation ratio $K_{\lambda }$ using the present correlation in Equation (11).

Figure 13 shows the ratio of change in the relaxation time $K_{\lambda }$, calculated with Equation (11) under three constant shear rate conditions ${\dot{\gamma }}_{mc}$= 10,000, 60,000 and 100,000 1/s. The solid and broken lines express the calculations for PAM 0.1 and PAM 0.3 solutions, respectively. The calculated values of $K_{\lambda }$ increased with the increasing of both the channel length *L* and the shear rate in the microchannel ${\dot{\gamma }}_{mc}$. Even without using a relaxation time measuring device, Equation (11) is useful for easily estimating the change in relaxation time when a PAM aqueous solution is flowed through a microchannel.

Studies on polymer solution in drag reduction have shown that different types of polymers have different degradation trends with a fixed high shear rate [50,51,52]. The proposed model, Equation (11), is based on experimental data for PAM aqueous solutions, so it is necessary to investigate whether the empirical constant *B* in the equation can be applied to other aqueous polymer solutions.

## 4. Conclusions

This paper investigated experimentally the change in rheological properties, i.e., viscosity and relaxation time, of a polyacrylamide aqueous solution after passing through a square rectangular microchannel with its different length. The experimental results showed that the viscosity and relaxation time of the solution decreased with the increasing of both the shear rate and the length in the microchannel. From the experimental results of viscosity and relaxation time, in the shear rate range (0 to 10,000 1/s), the degree of degradation increases rapidly with the increase in shear rate. Based on the experimental results, we proposed an empirical model to predict the ratio of the relaxation times before and after passing through the microchannel. The present results will be useful to estimate the change in rheological properties passed through the small size channel, and will contribute to industrial application such as development and design of micro-reactors and biochips.

## Figures and Tables

**Table 1:** Fitting parameter of power-law model given in Equation (6) for test liquids after flow through microchannel with various ${\dot{\gamma }}_{mc}$.

${\dot{\gamma }}_{\mathrm{mc}}$	PAM 0.1–100 mm		PAM 0.3–100 mm		PAM 0.1–200 mm		PAM 0.3–200 mm	
1/s	K [mPa· $s^{n}$]	n [−]	K [mPa· $s^{n}$]	n [−]	K [mPa· $s^{n}$]	n [−]	K [mPa· $s^{n}$]	n [−]
0	2.98	0.952	3.96	0.919	2.98	0.919	3.96	0.919
6000	2.47	0.927	2.39	0.947	2.55	0.928	2.40	0.911
10,000	2.49	0.931	2.39	0.940	2.57	0.922	2.40	0.93
20,000	2.43	0.951	2.43	0.947	2.61	0.912	2.39	0.923
40,000	2.40	0.922	2.40	0.958	2.66	0.926	2.42	0.931
60,000	2.49	0.925	2.45	0.956	2.65	0.936	2.42	0.931
80,000	2.61	0.967	2.48	0.966	2.69	0.940	2.44	0.939
100,000	2.60	0.954	2.53	0.971	2.71	0.946	2.51	0.944

**Table 2:** The relaxation time $\lambda_{o}$ (${\dot{\gamma }}_{mc}$ = 0) and $\lambda_{D}$ captured from the extensional elasto-capillary thinning measurement. (unit: ms).

Shear Rate (1/s)	PAM 0.1		PAM 0.3	
${\dot{\gamma }}_{\mathrm{mc}}$	100 mm	200 mm	100 mm	200 mm
0	7.38	7.38	7.72	7.72
6000	4.81	5.39	7.58	5.59
10,000	4.13	4.44	4.99	5.27
20,000	3.82	3.75	4.82	4.37
40,000	3.51	2.75	4.12	3.30
60,000	2.59	2.52	3.31	2.67
80,000	2.38	2.09	2.66	2.02
100,000	2.08	1.75	1.92	1.56

## Data Availability

The original contributions presented in the study are included in the article, further inquiries can be directed to the corresponding author.
